# The interaction between pain and attractiveness perception in others

**DOI:** 10.1038/s41598-020-62478-x

**Published:** 2020-03-26

**Authors:** Jing Meng, Xiong Li, Weiwei Peng, Zuoshan Li, Lin Shen

**Affiliations:** 1 Key Laboratory of Applied Psychology, Chongqing Normal University, Chongqing, China; 2 School of Education, Chongqing Normal University, Chongqing, China; 3 School of Psychology, Shenzhen University, Shenzhen, China; 4 Shenzhen Key Laboratory of Affective and Social Cognitive Science, Shenzhen University, Shenzhen, China; 5 School of Mathematical Sciences, Chongqing Normal University, Chongqing, China

**Keywords:** Electroencephalography - EEG, Empathy, Human behaviour

## Abstract

When considering the “beauty-is-good” stereotype, facial attractiveness should facilitate empathy for pain. On the other hand, having in mind the “threat value of pain” hypothesis, pain cues would be more salient, and thus, its processing would not suffer influence by facial attractiveness. The event-related potential (ERP) allows investigating if one of these theories could predict individuals’ responses regarding the perception of pain or attractiveness in others’ faces. We tracked 35 participants’ reactions to pictures depicting more and less attractive faces displayed in a painful and non-painful condition. Each participant completed the following two tasks when presented the images of faces: (1) the Pain Judgment Task, in which participants should rate the pain levels, and (2) the Attractiveness Judgment Task, in which participants should rate the attractiveness. Results showed that participants exhibited differences rating more and less attractive faces in the non-painful pictures, but not in the painful pictures. These results were observed in P3 and LPC amplitudes in the Pain Judgment Task, as well as in N170 and P2 amplitudes in the Attractive Judgment Task. Our results suggested that both explicit and implicit empathic pain processing inhibited the processing of attractiveness perception. These findings supported the “threat value of pain” hypothesis. Besides, in the Attractive Judgment Task, the N170 and P2 amplitudes for more attractive painful pictures were larger than those for more attractive non-painful pictures. In contrast, no significant difference was found between the amplitudes for painful and non-painful, less attractive pictures. Our findings suggest that explicit facial attractiveness processing for more attractive face images potentiates the implicit empathy for pain processing, therefore partly supporting the “beautiful-is-good” stereotype.

## Introduction

Empathy refers to the ability to understand and share others’ feelings^1^. When individuals witness others’ pain or injuries, they usually can identify the pain and relate to these experiences as if they were their own^2^. This competency is thus called empathy for pain^3,4^. According to the “threat value of pain” hypothesis^5–8^, the perception of another’s pain activates the individual’s survival mechanisms, meaning that, they may experience withdrawal and avoidance like when exposed to real or perceived danger. Therefore, the ability to recognize others’ pain helps individuals to avoid possible hazards and promotes empathic behavior^9^.

Physical attractiveness, as a symbol of biological quality signaling fertility and health^10,11^, plays an important role in interpersonal interactions in daily life. Attractive faces can be considered rewarding stimuli, eliciting positive emotional reactions. Those activate brain areas associated with the processing of positive emotions and rewarding, such as the nucleus accumbens and the ventral tegmental area^12^. According to the “beauty-is-good” stereotype^13^, people with higher physical attractiveness are deemed by others as possessing positive personality attributions, such as sociability, intelligence, and morality^14,15^.

One study showed that physical attractiveness facilitates empathic responses, observing a greater empathic response to pain in more attractive people, as compared to pain in less attractive people^16^. However, another study revealed that physical attractiveness inhibited empathy for children in need, especially when a victim’s need was obviously severe^17^. A functional magnetic resonance imaging study^18^ indicated that the modulation of physical attractiveness on pain empathy varied between male and female participants: there were greater activations in regions associated with empathy for pain processing, such as the insula and anterior cingulate cortex, to less attractive men and to more attractive women.

On the other hand, whether the perception of physical attractiveness in others could be influenced by pain remains unclear. Previous studies have shown that perceptions of facial attractiveness could be influenced by emotions in the faces, such that negative emotions suppressed attractiveness assessments as compared to positive and neutral emotional faces^19,20^. According to the “threat value of pain” hypothesis, others’ pain, serving as warning signals to avoid or escape^5–8^, would elicit negative emotional reactions^21–24^, thus suppressing the perception of physical attractiveness, as evidenced by decreased attractiveness ratings and neural responses.

Both others’ pain (signaling threat and danger) and attractiveness (signaling fertility and health) could be processed prioritized by the human attention system^25,26^. The present study aimed to explore the interplay between others’ pain and physical attractiveness as perceived by participants. More specifically, we examined (1) whether empathy for pain can be influenced by others’ attractiveness and (2) whether perceptions of others’ attractiveness can be influenced by others’ pain. Therefore, four categories of facial stimuli were adopted, i.e., non-painful and painful facial stimuli with either low or high physical attractiveness. Each participant completed two tasks. In the first, we applied the Pain Judgment Task, in which participants were asked to judge whether facial stimulation was painful or non-painful. In this way, others’ pain would be processed explicitly, while attractiveness would be processed implicitly. The second task consisted of the Attractiveness Judgment Task, in which participants were asked to assess whether the presented face was attractive or non-attractive. In this task, attractiveness processing would be explicit processed and pain processing would be implicit.

As predicted by the “beauty-is-good” stereotype, more attractive faces are perceived as being morally good and as having better personalities^13^, thus capturing more attention resources. Therefore, we hypothesized that both of the explicit and implicit processing of others’ pain would be potentiated by physical attractiveness processing, as reflected by heightened empathic neural responses to faces with high physical attractiveness. As predicted by the “threat value of pain” hypothesis, the perception of others’ pain and injure is a potential threat to the self, apparently provoking observers’ threat-detection system and possibly activating a general aversive response^7^. Therefore, we hypothesized that both of the explicit and implicit processing of others’ attractiveness would be suppressed by processing of others’ pain, as reflected by the decreased processing of facial attractiveness caused by pain cues present in the faces.

## Results

### Subjective ratings to facial stimuli

**Figure 1:**
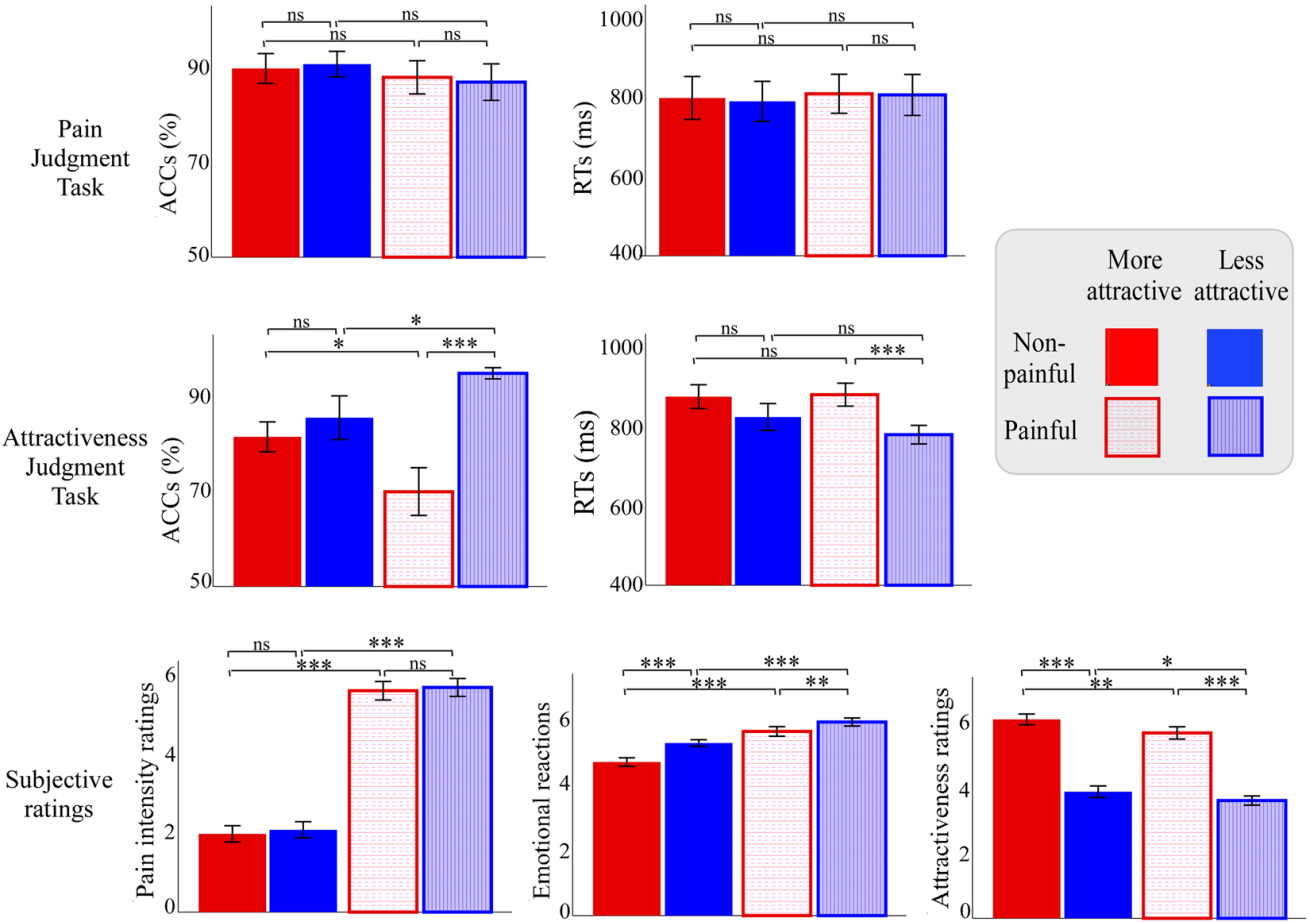
Behavioral responses to more and less attractive faces in painful and non-painful situations. RTs and ACCs in the Pain Judgment Task (top panel) and Attractiveness Judgment Task (middle panel), as well as subjective ratings (bottom panel) to non-painful (solid bar) and painful (dotted bar) pictures with high (red) and low (blue) attractiveness. Data in the bar chart were expressed as Mean ± SEM. ns: *p* > 0.05; *p < 0.05, **p < 0.01, ***p < 0.001.

As shown in the bottom panel of Fig. 1, pain intensity ratings were modulated by “pain” (*F*_*1,34*_ = 105.18, *p* < 0.001, *η*_*p*_^2^ = 0.76), with participants accurately judging painful and non-painful pictures (Painful: 5.60 ± 1.36 vs. Non-painful: 2.02 ± 1.21). Nevertheless, pain intensity ratings were modulated neither by “attractiveness” nor by the interaction (*p* > 0.05 for both comparisons).

The attractiveness ratings were significantly modulated by “attractiveness” (*F*_*1,34*_ = 108.90, *p* < 0.001, *η*_*p*_^2^ = 0.76) and by “pain” (*F*_*1,34*_ = 8.37, *p* = 0.007, *η*_*p*_^2^ = 0.20), indicating that participants judged more attractive models as more attractive than less attractive models (More attractive: 5.86 ± 1.04 vs. Less attractive: 3.73 ± 0.93), non-painful pictures as more attractive than painful pictures (Non-painful: 4.97 ± 1.01 vs. Painful: 4.62 ± 0.97).

Participants’ emotional reactions were modulated by “attractiveness” (*F*_*1,34*_ = 19.09, *p* < 0.001, *η*_*p*_^2^ = 0.36) and “pain” (*F*_*1,34*_ = 25.32, *p* < 0.001, *η*_*p*_^2^ = 0.43), indicating that the participants felt negatively toward the less attractive models, relative to the more attractive models (Less attractive: 5.58 ± 0.66 vs. More attractive: 5.14 ± 0.82), and felt negatively toward the painful pictures, relative to the non-painful pictures (Painful: 5.75 ± 0.80 vs. Non-painful: 4.97 ± 0.68). Emotional reactions were also significantly modulated by the interaction between “attractiveness” and “pain” (*F*_*1,34*_ = 6.86, *p* = 0.013, *η*_*p*_^2^ = 0.17), indicating that (1) the difference between less and more attractive models was greater for non-painful pictures than for painful pictures (Non-painful: 0.58 ± 0.75 vs. Painful: 0.29 ± 0.58; *F*_*1,34*_ = 6.86, *p* = 0.013, *η*_*p*_^2^ = 0.168), and that (2) the difference between painful and non-painful pictures was larger for more attractive models than for less attractive models (More attractive: 0.93 ± 1.14 vs. Less attractive: 0.64 ± 0.79; *F*_*1,34*_ = 6.86, *p* = 0.013, *η*_*p*_^2^ = 0.17).

### Pain judgment task

As shown in the top panel of Fig. 1, we did not observe a significant main effect or interaction effect in RTs and ACCs (*p* > 0.05 for all comparisons).

**Figure 2:**
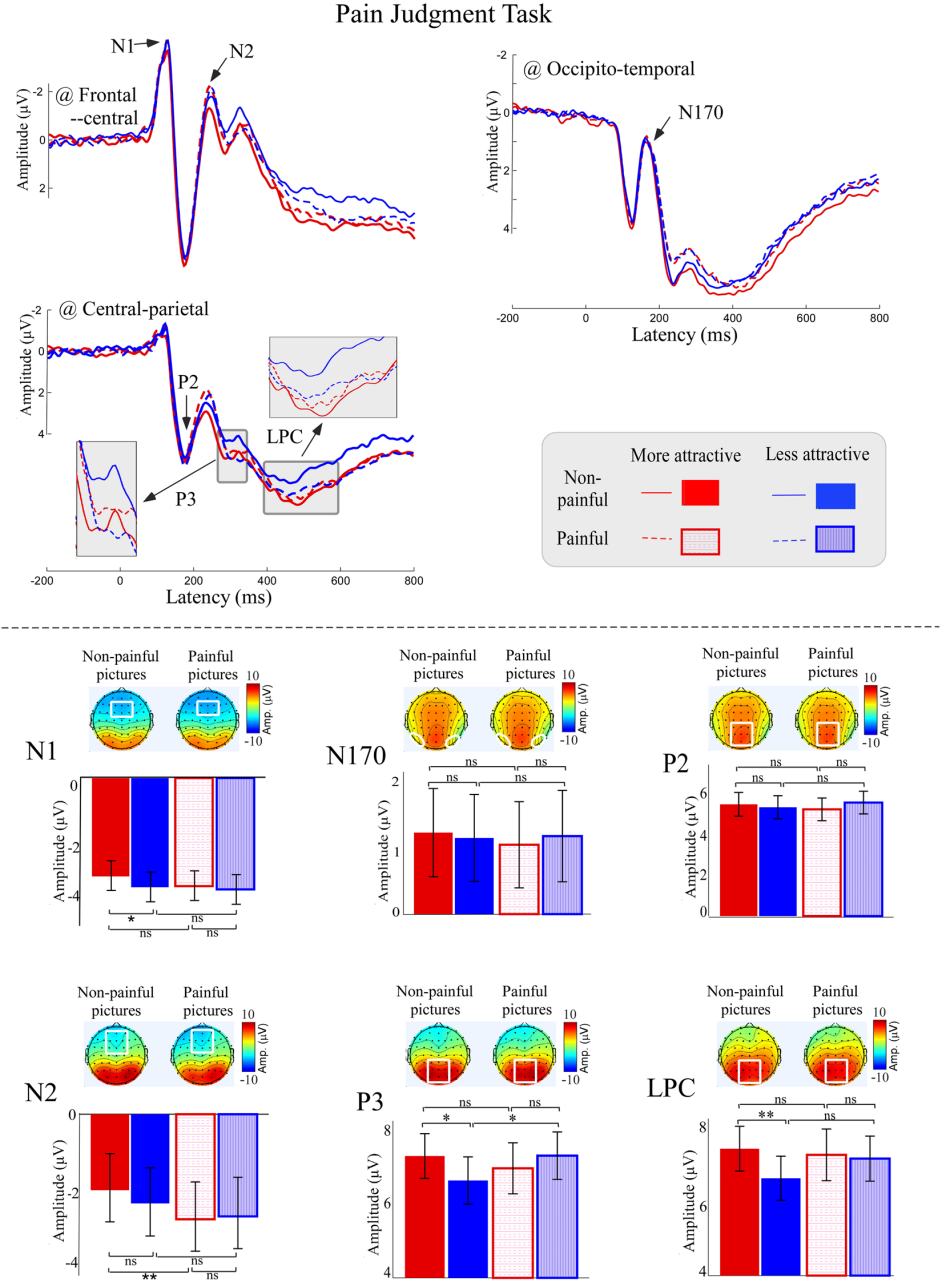
ERP responses to painful and non-painful pictures with high and low attractiveness in the Pain Judgment Task. ERP waveforms (top panel), bar charts and scalp topography distributions (bottom panel) elicited by non-painful (solid) and painful (dotted) pictures with high (red) and low (blue) attractiveness. Electrodes used to estimate ERP amplitudes were marked using the white squares on their respective topographic distributions. Data in the bar chart were expressed as Mean ± SEM. ns: *p* > 0.05; *p < 0.05, **p < 0.01, ***p < 0.001.

Grand average ERP waveforms to painful and non-painful pictures with high and low attractiveness in the Pain Judgment Task, as well as scalp topographies of dominant waves, are shown in Fig. 2. Regardless of whether painful or attractive, others’ faces elicited N1 and N2 waves over frontal-central electrodes (e.g., FCz and Fz), N170 waves over occipito-temporal electrodes (e.g., PO7 and PO8) and P2, P3 and LPC waves at central-parietal electrodes (e.g., CPz and Pz). Amplitudes of dominant waves in different conditions were compared using a repeated measures two-way ANOVA with factors of “attractiveness” (more vs. less) and “pain” (painful vs. non-painful), and relevant results have been summarized in Table 1.

**Table 1:** Summary of statistical analysis of neural responses to facial stimuli in the pain judgment task and in the attractiveness judgment task.

	Pain			Attractiveness			Attractiveness × Pain		
	*F*	*p*	*η*_*p*_^2^	*F*	*p*	*η*^2^	*F*	*p*	*η*_*p*_^2^
**Pain Judgment Task**									
**N1**	1.349	0.254	0.038	**4.774**	**0.036**	**0.123**	0.774	0.385	0.022
**N170**	0.182	0.673	0.005	0.053	0.819	0.002	0.510	0.480	0.015
**P2**	0.002	0.961	<0.001	0.345	0.561	0.010	3.973	0.054	0.105
**N2**	**5.393**	**0.026**	**0.137**	0.481	0.493	0.014	1.817	0.187	0.051
**P3**	0.335	0.566	0.010	0.628	0.434	0.018	**8.675**	**0.006**	**0.203**
**LPC**	0.288	0.595	0.008	2.833	0.102	0.077	**4.509**	**0.041**	**0.117**
**Attractiveness Judgment Task**									
**N1**	0.471	0.497	0.014	0.110	0.743	0.003	0.330	0.569	0.010
**N170**	1.310	0.260	0.037	**17.134**	**<0.001**	**0.335**	**8.021**	**0.008**	**0.191**
**P2**	0.455	0.505	0.013	**17.572**	**<0.001**	**0.341**	**6.549**	**0.015**	**0.162**
**N2**	**6.757**	**0.014**	**0.166**	0.001	0.982	<0.001	0.318	0.577	0.009
**P3**	**7.194**	**0.011**	**0.175**	0.065	0.801	0.002	1.249	0.272	0.035
**LPC**	0.914	0.346	0.026	**5.153**	**0.030**	**0.132**	0.051	0.822	0.002

Notes: Statistics were obtained using repeated measures ANOVA with within-participant of “attractiveness” and “pain” in the Pain Judgment Task and Attractiveness Judgment Task. df:(1,34) Significant comparisons (*p* < 0.05) were shown in boldface.

N1 amplitudes were significantly modulated by “attractiveness” (*F*_*1,34*_ = 4.77, *p* = 0.036, *η*_*p*_^2^ = 0.11), with N1 waves showing more negativity toward the less attractive faces than toward the more attractive faces (Less attractive: −3.84 ± 3.82 μV vs. More attractive: −3.60 ± 3.80 μV). N2 amplitudes were significantly modulated by “pain” (*F*_*1,34*_ = 3.39, *p* = 0.026, *η*_*p*_^2^ = 0.14), indicating that painful pictures elicited significantly greater N2 amplitudes than non-painful pictures (Painful: −2.62 ± 5.19 μV vs. Non-painful: −2.09 ± 5.15 μV).

P3 and LPC amplitudes were significantly modulated by the interaction between “attractiveness” and “pain” (P3: *F*_*1,34*_ = 8.66, *p* = 0.006, *η*_*p*_^2^ = 0.20; LPC: *F*_*1,34*_ = 4.51, *p* = 0.041, *η*_*p*_^2^ = 0.12). Although for the non-painful pictures, P3 and LPC amplitudes to the more attractive faces were larger than to the less attractive faces (P3: More attractive non-painful: 7.27 ± 3.55 μV vs. Less attractive non-painful: 6.61 ± 3.75 μV, *F*_*1,34*_ = 6.47, *p* = 0.016, *η*_*p*_^2^ = 0.16; LPC: More attractive non-painful: 7.40 ± 3.55 μV vs. Less attractive non-painful: 6.61 ± 3.50 μV, *F*_*1,34*_ = 8.08, *p* = 0.008, *η*_*p*_^2^ = 0.19), no significant difference of P3 and LPC amplitudes was observed between the more attractive and less attractive faces for the painful pictures (P3: More attractive painful: 6.94 ± 4.05 μV vs. Less attractive painful: 7.28 ± 3.77 μV, *F*_*1,34*_ = 1.61, *p* = 0.213, *η*_*p*_^2^ = 0.05; LPC: More attractive painful: 7.24 ± 4.10 μV vs. Less attractive painful: 7.14 ± 3.57 μV, *F*_*1,34*_ = 0.09, *p* = 0.769, *η*_*p*_^2^ < 0.01). Another direction of comparison of P3 amplitudes revealed that, for the less attractive faces, P3 amplitudes to the painful pictures were significantly larger than to the non-painful pictures (P3: Less attractive painful: 7.28 ± 3.77 μV vs. Less attractive non-painful: 6.61 ± 3.75 μV, *F*_*1,34*_ = 4.84, *p* = 0.035, *η*_*p*_^2^ = 0.13), whereas no significant difference was observed between the painful and non-painful pictures for the more attractive faces (P3: More attractive painful: 6.94 ± 4.05 μV vs. More attractive non-painful: 7.27 ± 3.55 μV, *F*_*1,34*_ = 0.81, *p* = 0.374, *η*_*p*_^2^ = 0.02).

### Attractiveness judgment task

As shown in the middle panel of Fig. 1, ACCs and RTs were modulated by “attractiveness” (ACC: *F*_*1,34*_ = 17.27, *p* < 0.001, *η*_*p*_^2^ = 0.34; RT: *F*_*1,34*_ = 8.65, *p* = 0.006, *η*_*p*_^2^ = 0.20), where participants judged the more attractive faces with less speed and accuracy than the less attractive faces (ACC: More attractive: 75.53 ± 24.88% vs. Less attractive: 89.89 ± 16.97%; RT: More attractive: 885.91 ± 173.77 ms vs. Less attractive: 800.03 ± 169.46 ms).

ACCs were significantly modulated by the interaction between “attractiveness” and “pain” (*F*_*1,34*_ = 5.33, *p* = 0.027, *η*_*p*_^2^ = 0.14). For the painful pictures, ACCs to the more attractive faces were lower than to the less attractive faces (More attractive painful: 69.82 ± 29.30% vs. Less attractive painful: 94.53 ± 6.93%, *F*_*1,34*_ = 22.14, *p* < 0.001, *η*_*p*_^2^ = 0.39), whereas no significant difference was observed between the more attractive and less attractive faces for non-painful pictures (More attractive non-painful: 81.24 ± 18.45% vs. Less attractive non-painful: 85.25 ± 27.00%, *F*_*1,34*_ = 0.44, *p* = 0.512, *η*_*p*_^2^ = 0.01). These results suggest that participants tended to judge more attractive faces with pain cues as less attractive.

**Figure 3:**
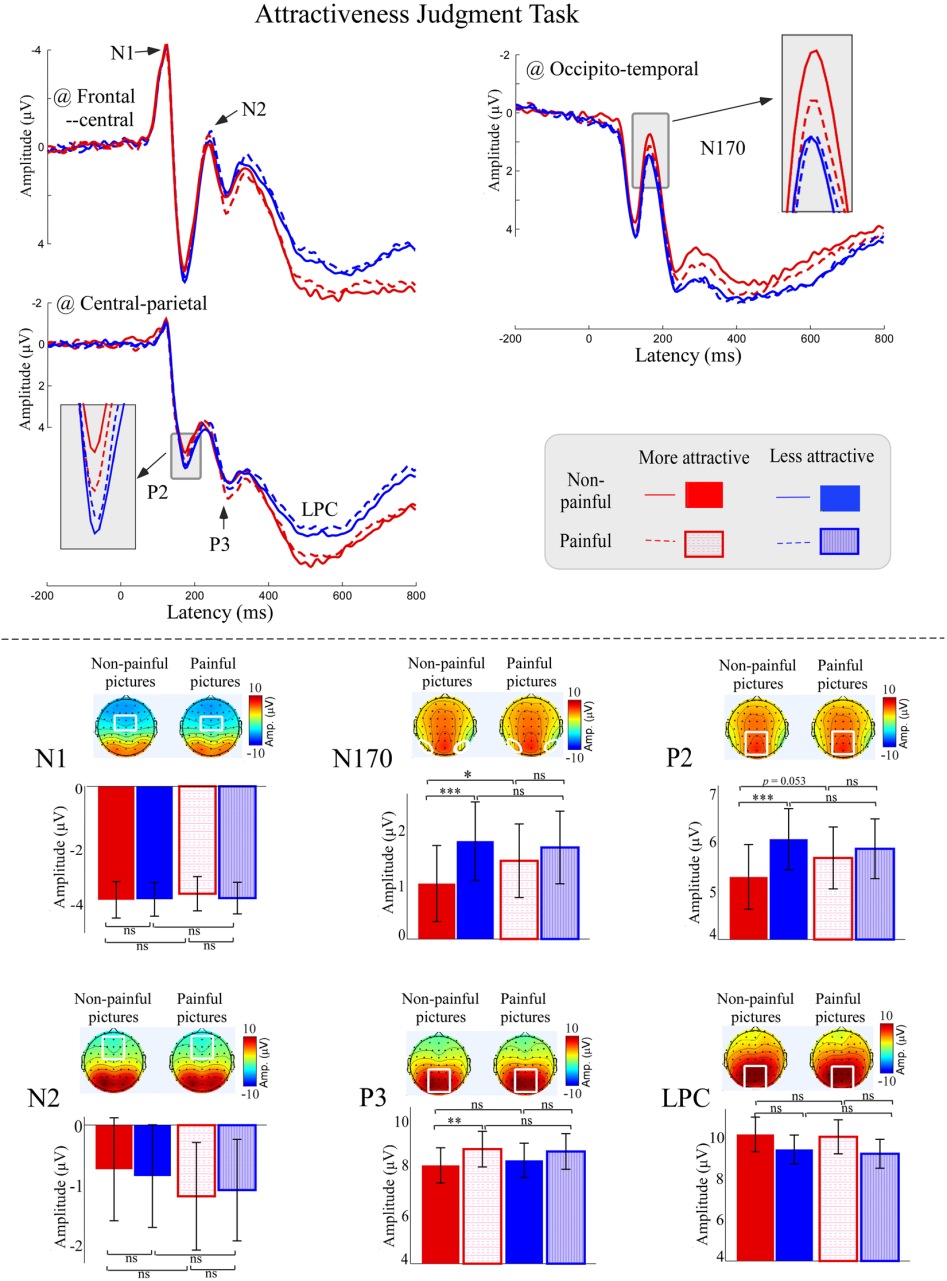
ERP responses to more and less attractive pictures with non-painful and painful cues in the Attractive Judgment Task. ERP waveforms (top panel), bar charts and scalp topographies (bottom panel) elicited by the observation of more (red) and less (blue) attractive pictures either with non-painful (solid) or painful (dotted) cues. Electrodes used to estimate the mean ERP amplitudes were marked using the white squares on their respective topographic distributions. Data in the bar chart were expressed as Mean ± SEM. ns: *p* > 0.05; *p < 0.05, **p < 0.01, ***p < 0.001.

Averaged ERP waveforms and scalp topographies of each condition are shown in Fig. 3. A full list of all statistical comparisons of ERP amplitudes can be found in Table 1. N170 and P2 amplitudes were modulated by “attractiveness” (N170: *F*_*1,34*_ = 17.13, *p* < 0.001, *η*_*p*_^2^ = 0.34; P2: *F*_*1,34*_ = 17.57, *p* < 0.001, *η*_*p*_^2^ = 0.34), with amplitudes being more negative to the more attractive faces than to the less attractive faces (N170: More attractive: 1.21 ± 4.01 μV vs. Less attractive: 1.72 ± 4.05 μV; P2: More attractive: 5.48 ± 3.84 μV vs. Less attractive: 5.96 ± 3.67 μV). N170 and P2 amplitudes were also significantly modulated by the interaction between “attractiveness” and “pain” (N170: *F*_*1,34*_ = 8.02, *p* = 0.008, *η*_*p*_^2^ = 0.19; P2: *F*_*1,34*_ = 6.55, *p* = 0.015, *η*_*p*_^2^ = 0.16). For the non-painful pictures, amplitudes to the more attractive faces were more negative than to the less attractive faces (N170: More attractive non-painful: 1.00 ± 4.08 μV vs. Less attractive non-painful: 1.77 ± 4.21 μV, *F*_*1,34*_ = 28.05, *p* < 0.001, *η*_*p*_^2^ = 0.46; P2: More attractive non-painful: 5.28 ± 3.93 μV vs. Less attractive non-painful: 6.06 ± 3.71 μV, *F*_*1,34*_ = 26.73, *p* < 0.001, *η*_*p*_^2^ = 0.44), whereas no significant difference was observed between the more attractive and less attractive faces for the painful pictures (N170: More attractive painful: 1.42 ± 3.95 μV vs. Less attractive painful: 1.65 ± 3.89 μV, *F*_*1,34*_ = 2.21, *p* = 0.146, *η*_*p*_^2^ = 0.06; P2: More attractive painful: 5.67 ± 3.76 μV vs. Less attractive painful: 5.86 ± 3.63 μV, *F*_*1,34*_ = 1.16, *p* = 0.289, *η*_*p*_^2^ = 0.03). Another direction of comparison revealed that, for the more attractive faces, amplitudes to the painful pictures were significantly or marginally larger than to the non-painful pictures (N170: More attractive painful: 1.42 ± 3.95 μV vs. More attractive non-painful: 1.00 ± 4.08 μV, *F*_*1,34*_ = 5.58, *p* = 0.021, *η*_*p*_^2^ = 0.14; P2: More attractive painful: 5.67 ± 3.76 μV vs. More attractive non-painful: 5.28 ± 3.93 μV, *F*_*1,34*_ = 4.02, *p* = 0.053, *η*_*p*_^2^ = 0.11), whereas no significant difference was observed between the painful and non-painful pictures for the less attractive faces (N170: Less attractive painful: 1.66 ± 3.89 μV vs. Less attractive non-painful: 1.77 ± 4.21 μV, *F*_*1,34*_ = 0.60, *p* = 0.445, *η*_*p*_^2^ = 0.02; P2: Less attractive painful: 5.86 ± 3.63 μV vs. Less attractive non-painful: 6.06 ± 3.71 μV, *F*_*1,34*_ = 1.31, *p* = 0.261, *η*_*p*_^2^ = 0.04).

N2 and P3 amplitudes were significantly modulated by “pain” (N2: *F*_*1,34*_ = 6.76, *p* = 0.014, *η*_*p*_^2^ = 0.17), with painful pictures eliciting significantly greater amplitudes than non-painful pictures (N2: Painful: −1.06 ± 4.81 μV vs. Non-painful: −0.74 ± 4.73 μV; P3: Painful: 8.57 ± 4.25 μV vs. Non-painful: 8.07 ± 4.14 μV). LPC amplitudes were only modulated by “attractiveness” (*F*_*1,34*_ = 5.15, *p* = 0.030, *η*_*p*_^2^ = 0.13), such that LPC amplitude was higher for the more attractive faces than for the less attractive faces (More attractive: 10.08 ± 4.92 μV vs. Less attractive: 9.31 ± 4.14 μV).

## Discussion

This study investigated interactions between the perception of pain and attractiveness in facial images. Our results showed that neither the explicit or the implicit empathic pain processing suffered influence by attractiveness, thus supporting the “threat value of pain” hypothesis^7^. Also, the explicit facial attractiveness processing for more attractive faces enhanced the implicit empathy for pain processing, therefore, partly supporting the “beautiful-is-good” stereotype^13^.

### Pain judgment task

In line with previous studies showing that painful stimuli elicited larger amplitudes relative to non-painful stimuli in the N2 time window^9,27^, the present study showed enlarged N2 amplitudes to painful pictures relative to non-painful pictures. The N2 amplitudes to others’ painful faces were suggested to be related to affective components of empathy for pain^28^. Thus, our findings suggest that emotional resonance was elicited to others’ facial pain in the present study.

We observed significant P3 amplitude differences between painful and non-painful pictures of less attractive models, which is consistent with findings of previous ERP studies on empathy for pain. Those studies have found larger amplitudes in the processing of painful images compared to non-painful, using images of hands and feet (see review^1^), and faces^29^. Interestingly, this effect was not observed when assessing more attractive faces. One possible explanation for those findings would be that the implicit attractiveness processing of more attractive faces could attenuate the empathic responses to others’ pain. Therefore, when individuals pay attention to the pain cues, empathy for pain could be reduced towards people with more attractive faces. Previous studies had found that empathic responses to others’ facial pain might be sensitive to some physical features, for instance, when participants assessed images of faces ethnically different than themselves, their empathy for pain was reduced^29,30^. The same happened regarding tasks involving trustworthy and untrustworthy faces^31^. It might be possible that own-race images or trustworthy faces are perceived as closer to oneself. Previous work has demonstrated that the perceived closeness to another individual enhances empathic responses^32^. Somehow, the more attractive models may be perceived as non-similar to the participants and the facial features may be more or less prioritized based on the implicit-explicit processing line of reasoning^33^. Thus, participants may exhibit inhibited empathic responses to more attractive models’ pain when they instructed to pay attention to the pain cues in the stimuli. However, we acknowledge that alternative explanations for these findings may be possible. As more attractive faces elicited larger P3 amplitudes than less attractive faces in later components^34^. Thus, enlarged P3 amplitudes for more attractive non-painful faces decreased the difference between painful and non-painful more attractive pictures. Above all, the results of the Pain Judgment Task suggest that explicit empathic processing to others’ facial pain inhibits the implicit attractiveness processing, which supports the “threat value of pain” hypothesis.

Most importantly, in the Pain Judgment Task, P3 and LPC amplitudes were significantly modulated by the interaction between “attractiveness” and “pain”. Differences between more and less attractive faces were only found in the non-painful pictures. Both behavioral results (ACCs, RTs, and pain intensity ratings) and ERP amplitudes to painful pictures were not influenced by the implicit attractiveness processing. Hence, these results suggest that explicit empathic processing to others’ facial pain inhibits the implicit attractiveness processing, which more supports the “threat value of pain” hypothesis^7^.

### Attractiveness judgment task

Consist with previous findings that individuals’ facial attractiveness was influenced by their expressions, negative emotions decreased perceptions of attractiveness^19,20^. Our findings demonstrated a significant effect on behavioral and neural responses of pain on attractiveness. Participants exhibited decreased accuracy in judging the more attractive painful pictures, suggesting that pain cues decrease the perception of attractiveness, especially to more attractive faces. In addition, attractiveness ratings to the more attractive faces showed a decrease in painful situations than did the less attractive models. These results indicated that pain decreased perceptions of attractiveness on a behavioral level.

In line with previous ERP studies^34,35^, N170, P2, and LPC amplitudes were modulated by “attractiveness”, with more attractive faces eliciting a negative deflection in N170 and P2 components and a positive deflection in LPC components relative to less attractive faces. Similarly to previous studies^9,27,29^, we also found a main effect of “pain” in N2 and P3 amplitude in the Attractive Judgment task, with painful pictures eliciting more negative N2 and more positive P3 waves than non-painful pictures. These results may suggest that N2 and P3 are sensitive to others’ pain cues, independently of task demands. As frontal N2 to others’ painful faces were suggested in relation to affective components of empathy for pain^28^, and as P3 over the posterior parietal cortical area have been thought to link to a cognitive evaluation component of empathy^29^, it appears that processing resources of evaluation of others’ pain were recruited automatically in these time windows even though the pain cues in the Attractive Judgment Task were unrelated to task goals.

More importantly, N170 and P2 components were modulated by the interaction between “pain” and “attractiveness”; that is, for the non-painful pictures, N170 and P2 waves to the more attractive faces were more negative than to the less attractive faces, whereas no difference was found between the more and less attractive faces for the painful pictures. Previous ERP findings^27,31^ suggested that the N170 and P2 components were involved in the automatic coding of both facial identity and emotional state, such as pain. Thus, our results suggested that implicit pain processing decreased the distinguishing automatic coding process of others’ attractiveness in these time windows, supporting the “threat value of pain” hypothesis^7^.

Interestingly, another aspect of the comparison between “pain” and “attractiveness” revealed that for more attractive faces, N170 and P2 amplitudes of painful pictures were larger than non-painful pictures, whereas no significant difference was observed between the painful and non-painful pictures for less attractive faces. These results suggest that explicit facial attractiveness processing for more attractive faces enhanced the implicit empathy for pain. Based on the “beauty-is-good” stereotype^13^, more attractive faces are perceived as being morally good and as having better personalities, thus, this would increase the observers’ empathy for pain. Under this perspective, our findings partly supported the “beauty-is-good” stereotype^13^.

### Interplay between processing others’ pain and attractiveness

The findings of both tasks in the present study indicate that empathy for pain and facial attractiveness may not be processed independently of each other. The perception of facial attractiveness could be suppressed by empathy for pain, regardless of the task, which strongly supports the “threat value of pain” hypothesis^7^. That is, both attractive faces^34,35^ and pain cues^36^ automatically capture our attention faster and hold it longer. When offered simultaneously, however, individuals would automatically allot more attention to pain cues. On the other hand, the explicit facial attractiveness processing for more attractive faces enhanced the implicit empathy for pain processing. These results partially support the “beauty-is-good” stereotype^13^.

Despite possible implications, several limitations of the present study should be addressed. Firstly, all face images were gray scale: whether these responses related to daily life requires further investigation. Secondly, both female and male prototypes were involved in the study: it may be that the effect is different for male and female faces. Future research should include gender in the experimental design.

## Conclusions

To investigate the association between empathy for pain and the attractiveness, this study employed the Pain Judgment Task and the Attractiveness Judgment Task use ERPs. The results suggested that both explicit and implicit empathic pain processing inhibited the processing of others’ attractiveness, thus supporting the “threat value of pain” hypothesis^7^. Furthermore, explicit facial attractiveness processing for more attractive faces enhanced the implicit empathy for pain processing, which partly supported the “beautiful-is-good” stereotype^13^.

## Materials and methods

### Participants

Thirty-five adults (18 females) from the Chongqing Normal University, Chongqing, China, participated in this study as paid volunteers. None of the participants had been previously diagnosed with a psychiatric, medical, or neurological disorder. All participants were right-handed, aged 18–24 years (Mean = 20.7 years, SD = 2.5 years), and in possession of normal or corrected-to-normal vision. All participants signed informed consent after receiving a complete description of the study. All participants gave their free and informed consent to the study before the experiment in accordance with the Declaration of Helsinki, and all procedures were approved by the Chongqing Normal University research ethics committee. The procedures were performed in accordance with ethical guidelines and regulations.

### Stimuli

**Figure 4:**
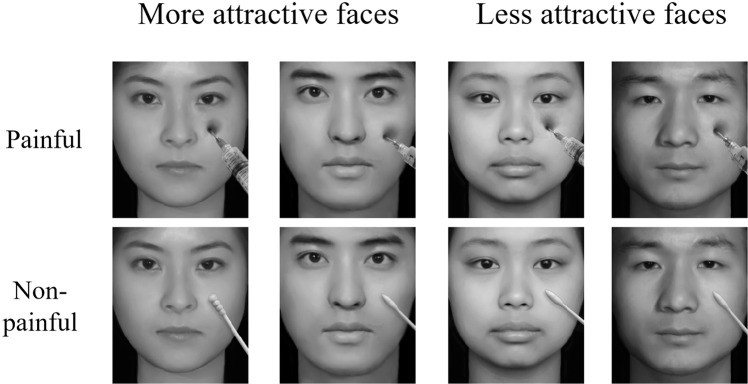
Examples of painful (top panel) and non-painful (bottom panel) pictures. Examples of more (left panel) and less (right panel) attractive pictures. Pictures were revised from a picture database that had been previously validated and used in published studies^37,38^.

The stimuli were 120 digital pictures of faces depicting painful and non-painful conditions, revised from a picture database that had been previously validated and used in published studies, in which the face images were morphed pictures^37,38^. The database comprised photos of 30 more attractive faces (15 female faces and 15 male faces) and 30 less attractive faces (15 female faces and 15 male faces). Each face was transformed to depict pain by penetrating the model’s cheek with a syringe needle, and a non-painful version was created by touching each model’s face with a soft object (Q-tip), using the software “Adobe Photoshop CS6” (Fig. 4). Luminance, contrast, and colour were matched between both groups of pictures.

Based on a 9-point Likert scale, pain intensity (1 = no sensation, 4 = pain threshold, 9 = unbearable pain), attractiveness levels (1 = not at all attractive, 9 = most attractive), emotional valence (1 = very unhappy, 9 = very happy), and arousal (1 = extremely peaceful, 9 = extremely exciting) of pictures were assessed by 42 undergraduate students (they did not participate in the experiment). In addition, emotional appearance of the faces (1 = very sad, 5 = neutral, 9 = very happy) were assessed by 30 undergraduate students (they did not participate in the experiment). The descriptive statistics and statistical analysis of pictures were summarized in Supplementary Material, S. Table 1.

### Experimental procedure

The participants were seated in a quiet room with an ambient temperature of about 20 °C. They were instructed to participate in two experimental tasks: (1) the Pain Judgment Task and (2) the Attractiveness Judgment Task. The order of these two tasks was counterbalanced between participants. For both tasks, the order of stimulus presentation was randomized, using the E-Prime (3.0) program. Electroencephalography (EEG) data were recorded during these two tasks.

**Figure 5:**
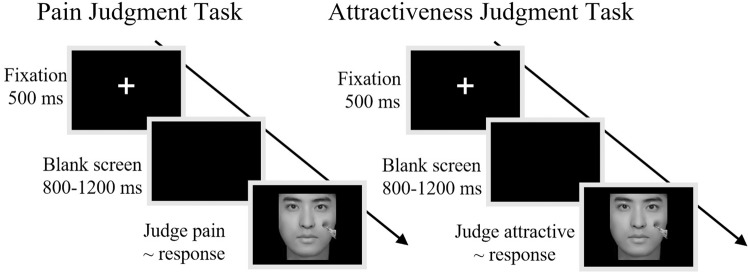
Flowchart describing the experimental design. Left column: Procedure of the Pain Judgment Task. Right column: Procedure of the Attractiveness Judgment Task.

In the Pain Judgment Task (shown in the left column in Fig. 5), participants were instructed to determine whether the model was experiencing pain. At the start of a Pain Judgment Task trial, a 500 ms fixation cross was presented on a black screen, followed 800–1,500 ms later by a picture, and the participants were instructed to respond as accurately and as quickly as possible by pressing a key (either “1” or “2”) to judge whether the picture depicted pain. The picture disappeared from the screen as soon as the participant responded. Key-pressing was counterbalanced across participants to control for order effects. The Pain Judgment Task comprised four blocks with 150 trials per block and an inter-trial interval of 1,000 ms. Each picture was presented five times during this task.

In the Attractiveness Judgment Task (shown in the right column in Fig. 5), participants were instructed to press a key (‘1’ or ‘2’), as accurately and as quickly as possible, to select whether the face was more attractive or less attractive. Except for different experimental instructions, procedures and stimuli in this task were identical to those in the Pain Judgment Task.

### Measurement of subjective reports

After the EEG recording session (Pain Judgment Task and Attractiveness Judgment Task), participants were instructed to rate the picture attributes of pain intensity (1 = no sensation, 4 = pain threshold, 9 = unbearable pain) and attractiveness levels (1 = not at all attractive, 9 = more attractive), as well as their subjective emotional reactions (1 = very happy, 9 = very unhappy), based on 9-point Likert scales. The experimental procedure of this task was illuminated in Supplementary Material, S. Figure 1. Each picture was presented one time during this task.

### EEG recording

EEG data were recorded from 64 scalp sites, using tin electrodes mounted on an actiCHamp system (Brain Vision LLC, Morrisville, NC, US; pass band: 0.01–100 Hz; sampling rate: 1000 Hz). The electrode at the right mastoid was used as a recording reference, and that on the medial frontal aspect was used as the ground electrode. All electrode impedances remained below 5 kΩ.

### EEG data analysis

EEG data were pre-processed and analyzed via MATLAB R2014a (MathWorks, USA) and the EEGLAB toolbox^39^. Continuous EEG signals were band-passed filtered (0.01–40 Hz) and segmented using a 1000 ms time window. Time windows of 200 ms before and 800 ms after the onset of stimuli were extracted from the continuous EEG, and the extracted window was baseline-corrected by the 200 ms time interval prior to stimuli onset. EEG epochs were baseline-corrected by a 200 ms time interval prior to stimuli onset. EEG epochs were also visually inspected, and trials containing significant noise from gross movements were removed. EOG artifacts were corrected via the independent component analysis (ICA) algorithm^40^. These epochs constituted 5 ± 2.4% of the total number of epochs.

After confirming scalp topographies in both single-participant and group-level ERP waveforms and previously reported studies^27,28,31^, dominant ERP components were identified as follows: N1 (FCz, FC1, FC2, Cz, C1 and C2); N2 (AFz, AF3, AF4, Fz, F1, F2, FCz, FC1 and FC2); P2, P3 and LPC (CPz, CP1, CP2, Pz, P1, P2, POz, PO3, and PO4); N170 (P7, P8, PO7 and PO8). Amplitudes of N1, N2, P2 and P3 components were calculated as the mean amplitudes with a latency interval of peak ± 10 ms at electrodes displaying maximal responses. In addition, LPC amplitudes were measured at latency intervals of 400–600 ms.

### Statistical analysis

Subjective ratings of pictures, including ratings of pain intensity, attractiveness and subjective emotional reaction, were compared using repeated measures analysis of variance (ANOVA) with factors of “attractiveness” (more attractive vs. less attractive) and “pain” (painful vs. non-painful). If any main effect or interaction effect was observed, post hoc comparisons were performed.

Behavioral data, including reaction times (RTs) and accuracies (ACCs), and electrophysiological data (peak latencies and amplitudes of dominant ERP components) were compared separately for the Pain Judgment task and for the Attractiveness Judgment task. Subjective ratings to stimuli, behavioral data and electrophysiological data were compared via a repeated measures ANOVA using the within-participants factors, “attractiveness” (more attractive vs. less attractive) and “pain” (painful vs. non-painful). If any main effect or interaction effect was observed, post hoc comparisons were performed.

In addition, a separate analysis with a factor for “order” (Pain Judgment Task First vs. Pain Judgment Task Second) was taken into account the possible order effect with which the tasks were performed. However, none significant comparisons were found in all dependent variables (all *p* > 0.05). Relevant results have been summarized in Supplementary materials, S. Table 2.

### Ethics approval and consent to participate

This research was approved by the Chongqing Normal University research ethics committee. All participants had signed informed consent after being given a complete description of the study. The ethics committee approved this consent procedure.

## Supplementary information

Supplementary materials.

## Data Availability

Supplementary data associated with this article can be found in the online version at https://pan.baidu.com/s/1ndSIVBg-gp_MiyCiqCmEfg.
