# Supplementary material for: The interaction between pain and attractiveness perception in others
Source: Sci Rep. 2020 Mar 26;10:5528. doi: 10.1038/s41598-020-62478-x (PMC7099075; doi:10.1038/s41598-020-62478-x)
Supplement: Supplementary file 1 — Supplementary materials. [file 41598_2020_62478_MOESM1_ESM.pdf]

Supplementary materials for "The interaction between pain and attractiveness perception in others"

Jing Meng[1,2,+], Xiong Li[1,2,+], Weiwei Peng[3, 4], Zuoshan Li[1,2], and Lin Shen[5*]

S. Table 1. Subjective ratings to different categories of facial stimuli (Mean ± SD)

	More attractive		Less attractive	
	**Painful**	**Non-painful**	**Painful**	**Non-painful**
**Pain intensity**	6.83 ± 0.20	6.77 ± 0.24	1.41 ± 0.13	1.50 ± 0.15
**Attractiveness**	6.05 ± 0.47	3.60 ± 0.29	6.14 ± 0.42	3.67 ± 0.50
**Emotional valence**	3.41 ± 0.16	3.17 ± 0.16	4.17 ± 0.23	3.39 ± 0.24
**Arousal**	2.95 ± 0.23	2.50 ± 0.17	3.10 ± 0.22	2.45 ± 0.18
**Emotional appearance**	4.78 ± 0.52	5.03 ± 0.25	4.67 ± 0.53	4.96 ± 0.21

Notes: Statistics were obtained using Paired-Samples T test of Emotional appearance with "attractive" (More attractive vs. Less attractive) for non-painful pictures. No significant difference was observed between the more attractive (5.03 ± 0.25) and less attractive (4.96 ± 0.21) non-painful pictures ($t(29)=2.015$, $p=0.053$).

S. **Table 2.** Summary of statistical analysis of the factor for “order”

	Pain Judgment Task Order	More attractive non-painful			More attractive painful			Less attractive non-painful			Less attractive painful		
		Mean±SD	*t*	*p*	Mean±SD	*t*	*p*	Mean±SD	*t*	*p*	Mean±SD	*t*	*p*
**Behavioral data**													
**Pain intensity ratings**	First	1.83±1.15	-0.6	0.4	5.42±1.42	-0.6	0.54	1.98±1.17	-0.	0.6	5.67±1.24	0.1	0.9
	Second	2.11±1.28	9	96	5.71±1.38	2	0	2.16±1.27	42	77	5.62±1.46	0	23
**Attractiveness ratings**	First	4.51±0.93	-1.3	0.1	5.51±1.00	-0.7	0.48	5.17±0.73	-0.	0.3	5.87±0.81	-0.	0.8
	Second	4.86±0.51	6	82	5.71±0.71	1	5	5.35±0.42	86	94	5.92±0.66	19	48
**Emotional reactions**	First	6.14±0.87	0.4	0.6	5.75±0.89	0.53	0.60	3.81±0.91	-0.	0.7	3.60±0.73	0.0	0.9
	Second	6.00±1.10	0	89	5.55±1.32		0	3.92±1.18	34	38	3.59±0.95	6	55
**RTs (ms)**	First	760.74±322.60	-0.6	0.4	777.90±278.07	-0.6	0.54	757.37±289.80	-0.	0.5	757.02±283.51	-0.	0.3
	Second	835.43±321.53	9	98	838.78±308.40	1	3	821.71±311.18	63	31	854.86±327.20	95	51
**ACCs**	First	0.91±0.16	0.7	0.4	0.88±0.22	-0.0	0.99	0.91±0.17	0.1	0.8	0.89±0.20	0.6	0.5
	Second	0.87±0.21	7	46	0.88±0.20	1	6	0.90±0.15	7	70	0.84±0.25	2	39
**ERP data**													
**N1**	First	-2.39±2.90	1.7	0.0	-3.09±3.31	1.05	0.30	-2.99±3.04	1.3	0.1	-2.94±2.95	1.4	0.1
	Second	-4.52±4.32	2	94	-4.48±4.40		0	-4.67±4.15	7	79	-4.87±4.67	7	51
**N170**	First	0.99±3.33	-0.3	0.7	0.76±3.45	-0.4	0.67	1.06±3.56	-0.	0.9	0.76±3.74	-0.	0.5
	Second	1.43±4.43	4	38	1.31±4.15	3	2	1.19±4.14	10	18	1.56±4.31	59	61
**P2**	First	5.29±3.26	-0.2	0.8	4.80±3.21	-0.7	0.47	5.13±3.24	-0.	0.7	5.08±3.25	-0.	0.4
	Second	5.59±3.69	5	03	5.60±3.38	2	6	5.47±3.48	30	64	5.99±3.32	82	18
**N2**	First	-2.17±5.00	-0.2	0.7	-3.25±4.62	-0.7	0.48	-2.94±4.69	-0.	0.4	-3.30±4.28	-0.	0.4
	Second	-1.65±5.62	9	73	-2.02±5.79	0	9	-1.53±5.47	82	19	-1.82±6.07	84	09
**P3**	First	7.31±4.05	0.0	0.9	6.51±4.59	-0.6	0.52	6.51±4.28	-0.	0.8	6.90±4.13	-0.	0.5
	Second	7.22±3.06	76	40	7.39±3.46	4	5	6.72±3.21	17	68	7.67±3.42	60	54
**LPC**	First	7.16±4.02	-0.4	0.6	6.84±4.85	-0.5	0.56	6.52±4.14	-0.	0.8	7.04±4.36	-0.	0.8
	Second	7.66±3.09	2	79	7.65±3.22	8	7	6.71±2.79	16	73	7.24±2.63	16	76

Notes: Statistics were obtained using Independent-Samples T test with the between-participant variable of “order” (Pain Judgment Task first vs. Pain Judgment second). None significant comparisons were found in all dependent variables (all $p > 0.05$). $N_{\text{Pain Judgment Task first}} = 18$, $N_{\text{Pain Judgment Task second}} = 17$, df:(33)

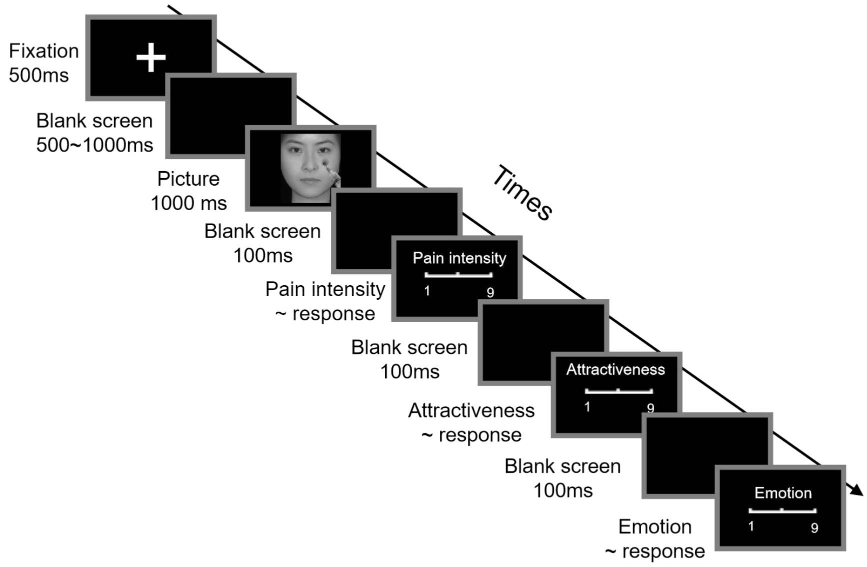

S. Figure 1. Flowchart describing the experimental design of measurement of subjective reports
